# Incorporation of Wheat Straw Ash as Partial Sand Replacement for Production of Eco-Friendly Concrete

**DOI:** 10.3390/ma14082078

**Published:** 2021-04-20

**Authors:** Shazim Ali Memon, Usman Javed, Muhammad Haris, Rao Arsalan Khushnood, Jong Kim

**Affiliations:** 1 Department of Civil and Environmental Engineering, School of Engineering and Digital Sciences, Nazarbayev University, Astana 010000, Republic of Kazakhstan; jong.kim@nu.edu.kz; 2 School of Civil and Mechanical Engineering, Curtin University, Perth, WA 6102, Australia; usman.javed1@postgrad.curtin.edu.au; 3 Department of Civil Engineering, COMSATS Institute of Information and Technology, Abbottabad 22060, Pakistan; mharis8408@gmail.com; 4 NUST Institute of Civil Engineering, National University of Sciences and Technology, Islamabad 44000, Pakistan; arsalan.khushnood@nice.nust.edu.pk

**Keywords:** aggregate resources, wheat straw ash, depletion, Chapelle activity, thermogravimetric analysis, pozzolanic hydrates, structural concrete

## Abstract

The depletion of natural sand resources occurs due to excessive consumption of aggregate for concrete production. Continuous extraction of sand from riverbeds permanently depletes fine aggregate resources. At the same time, a major ecological challenge is the disposal of agricultural waste ash from biomass burning. In this study, an environmental friendly solution is proposed to investigate the incorporation of wheat straw ash (WSA) by replacing 0, 5, 10, 15, and 20% of sand in concrete. Characterization results of WSA revealed that it was well-graded, free from organic impurities, and characterized by perforated and highly porous tubules attributed to its porous morphology. A decrease in fresh concrete density and an increase in slump values were attained by an increase in WSA replacement percentage. An increasing trend in compressive strength, hardened concrete density, and ultrasonic pulse velocity was observed, while a decrease was noticed in the values of water absorption with the increase in WSA replacement percentages and the curing age. The WSA incorporation at all replacement percentages yielded concrete compressive strength values over 21 MPa, which complies with the minimum strength requirement of structural concrete as specified in ACI 318-19. Acid resistance of WSA incorporated concrete improved due to the formation of pozzolanic hydrates as evident in Chappelle activity and thermogravimetric analysis (TGA) results of WSA modified composites. Thus, the incorporation of WSA provides an environmentally friendly solution for its disposal. It helps in conserving natural aggregate resources by providing a suitable alternative to fine aggregate for the construction industry.

## 1. Introduction

Human activities have spread chaos in the sustainability of planet Earth. It is evident from the current state of our surroundings that rapid environmental degradation and increased consumption of geological resources have caused its everlasting depletion. Concrete is an extensively utilized construction material in the world that requires an enormous amount of aggregate. Natural resources are being consumed by materials extraction. According to Wiedmann et al. [1], 69.68 billion tons of materials are extracted annually around the globe, in which construction materials and biomass contribute 29.58 and 20.29 billion tonnes/year respectively. Therefore, the natural aggregate sources are in a continuous state of depletion to fulfill the demand for concrete production.

According to statistics of the United Nations’ Food and Agricultural Organization, wheat produced globally was 743 million tons in 2017 [2]. It is estimated that between 1.3 to 1.4 kg of wheat straw is produced in the processing phase yielding one kilogram of wheat [3]. Wheat crop yields wheat straw as a byproduct when grains are separated from chaff and stalk. After separating grains, the field is being prepared for sowing the next crop by removing weeds through open field burning of crop residue, which disseminates nutrients to the soil [4]. Due to its high calorific value of 19.5 MJ/Kg [5], it is also used in industries as a source of energy production by burning [6,7]. The burning of wheat straw produces a large amount of waste ash dumped into ash ponds causes locking of the useful land [8], along with atmospheric degradation through the generation of particulate matter, and it poses several health hazards. Furthermore, as reported by WHO, in 2012, air pollution has caused 6.7% of deaths (3.7 million deaths) all over the world [9]. Due to the extremely high value of land especially in urban areas, the ash disposal process may become significantly uneconomical. The leachate problem associated with dumped ash poses a severe threat to water bodies that exist underground [10]. Furthermore, dumped ash accounts for air pollution and may have serious health hazards to human health. Being agricultural economies, India and China contribute 110Tg and 84Tg respectively of biomass [11]. According to Streets et al. [11], out of 730 Tg of biomass annually burned in Asia, 250 Tg (33.4%) involves an open-air burning of crop residue. Consequently, particulates, i.e., particles less than 2 mm (PM2.5), released during biomass burning, reduce visibility and degrade air quality, which causes deadly hazards to life after when generated at high levels on runways and highways, etc. [12].

Concrete usage has increased exponentially in the last three decades worldwide [13]. Being a prime constituent of concrete, aggregate demand has also increased at a similar pace, which has resulted in the depletion of the natural sand reservoir [14]. Due to increased population growth and advancement in infrastructure development, uncontrolled extraction of aggregates from the riverbed is a major concern [15], which may compromise the needs of the future generation. Therefore, constant efforts are employed to replace aggregate with artificial and recycled aggregate in concrete to fulfill the aggregate demand for achieving sustainable concrete [16,17,18,19,20]. Several studies were conducted to conserve natural fine aggregate by replacing it with agroindustrial waste ashes [21], including rice husk ash (RHA) [22] sugar cane bagasse ash [23], waste foundry sand, and bottom ash [24], as a partial sand substitute for the conservation of natural river sand resources.

Binici et al. [25] reported that the fresh and hardened properties of concrete improved upon increasing additives of corncob ash (CCA), wheat straw (WSA), and plane leaf ashes (PLA) by 2, 4, and 6% weight of fine aggregate, respectively. Kunchariyakun et al. [26] utilized RHA in autoclave aerated concrete as partial sand replacement. The results indicated that the reduction in autoclaving time and the temperature occurred upon the partial sand replacement with RHA in autoclave aerated concrete [26]. Samataray et al. [27] investigated the incorporation of RHA as partial fine aggregate replacement in conventional and self-compacting concrete. The results depicted that the improvement of compressive strength was observed for 30% and 20% replacement of RHA in normal and self-compacting concrete, respectively [27]. Singh and Siddique [28] justified the utilization of coal bottom ash as a fine aggregate substituent for the achievement of economical concrete as it increases the strength gain significantly and reduces permeable pore space along with the curing age. Siddique [29] also investigated fly ash usage (at 10, 20, 30, 40, and 50%) as fine aggregate replacement. The strength properties of concrete appeared to be improved compared to control concrete. Khushnood et al. [30] incorporated WSA and bentonite in self-compacted concrete as supplementary cementitious material and reported better mechanical and durability performance upon incorporation levels due to the formation of pozzolanic hydrates. A. Qudoos et al. [31] utilized polypropylene fiber and WSA in concrete as partial replacement of cement. A decrease in compressive strength and increasing flexure strength with the incorporation of polypropylene fibers were reported. However, WSA densified microstructure due to pozzolanic activity. Singh and Siddique [32] reported a maximum increase of 20% compressive strength at 40% substituent of fine aggregate with iron slag in SCC. Thus, SCC containing iron slag yielded the structural concrete. The authors [33] recommended the usage of fine and coarse brick replacement in concrete till 50% and 25% replacement levels respectively, which were investigated for mechanical and durability properties. Kou and Poon [34] investigated and justified the crushed fine stone, furnace bottom ash, and fine recycled aggregate as fine aggregate replacement in concrete. Finally, in recently published papers [15,28,35,36,37], the authors have identified agricultural and industrial wastes as a suitable option for sand replacement in concrete.

In short, researchers found the incorporation of agricultural and industrial waste suitable for the achievement of sustainable concrete as it has disposal problems and depletion of natural sand associated with it. It is worth mentioning that WSA has been previously used as a cement blend and composite [38,39,40]. The high concentration of amorphous silica in WSA induces pozzolanic reaction upon its incorporation in concrete [40], like that of other supplementary cementitious materials such as fly ash, ground granulated blast furnace slag and metakaolin as reported in the literature [41]. However, the current study aims to characterize the WSA at both macro- and microscopic levels for its successful incorporation in concrete as a partial sand replacement alongside evaluating its chemical reactivity that might contribute to its strength. The fresh, hardened and durability properties of concrete were investigated at various proportions of WSA. The incorporation of WSA in concrete has the potential to produce eco-friendly concrete with enhanced properties.

## 2. Materials and Methods

### 2.1. Raw Materials

Cement, sand, WSA, and coarse aggregate were used in this research. Type-I cement satisfying the requirements of ASTM standard C150 [42] along with the relative density and Blaine’s specific surface area values of 3.15 and 2786 cm^2^/g, respectively was used. Coarse aggregate was taken from the quarry site of Dorr River, Havelian (Pakistan) having a nominal maximum size of 19 mm, whereas Lawrencepur (Pakistan) river sand was used as fine aggregate. WSA was obtained from open-air burning (uncontrolled) of wheat straw, which was then used as a partial sand substituent in concrete.

### 2.2. Macro-Structural Characterization

**Figure 1:**
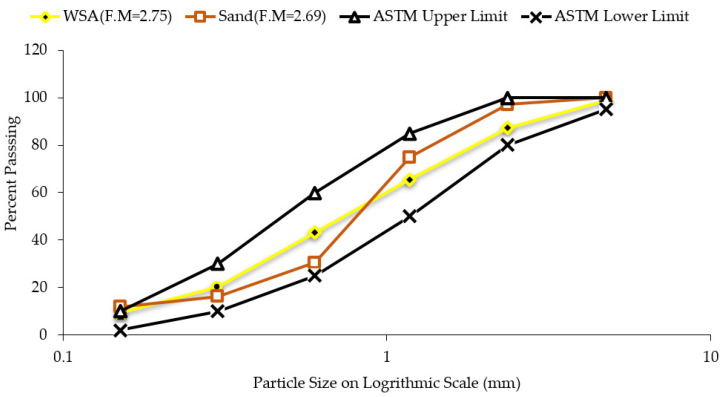
Gradation curves of sand and WSA along with ASTM C33 limits.

Characterization of aggregate was conducted at the macro level to assess its suitability as fine aggregate. At the macroscopic level, particle size distribution, water absorption, organic impurities of WSA, and aggregate were conducted as per ASTM standard C136 [43], C40 [44], and C128 [45], respectively. The results of particle size distribution revealed that WSA is well-graded, and it complies with both upper and lower limits specified in ASTM Specifications C33 [46] which is shown in Figure 1. The values of fineness modulus for sand and WSA were determined as 2.69 and 2.75, respectively. Due to the fibrous nature of WSA particles, its fineness modulus was higher than that of sand. The relative density of WSA is lesser than that of sand which is shown in Table 1. There are several chemical contaminants along with organic compounds such as monosaccharides and polysaccharides, humic acid, and lignin that can hinder the hydration reaction [47]. Therefore, an organic impurity test was performed, and it was revealed that the WSA is free from organic impurities. Usually, materials finer than 75 µm (#200) are responsible for increased affinity for water [48], the concentration of microfines was found to be 2.5% and 4.3%, respectively, whereas below 5% is the limit prescribed by ASTM specification C-33 [46]. Furthermore, the value of percentage passing on each sieve and percentage retained on the next consecutive sieve was determined less than 45%, fulfilling the requirement of ASTM C-33 [46]. Therefore, WSA complies with the standard requirements of particle size distribution. The relative density of WSA (1.89) reported in Table 1 was less than sand (2.62), which might change the hardened density of concrete while using it as a substituent of fine aggregate.

### 2.3. Microstructural Characterization

**Figure 2:**
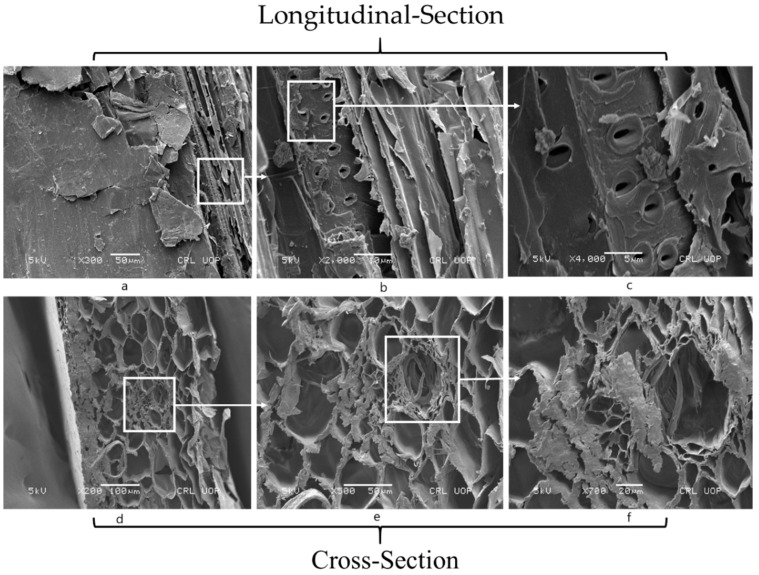
Morphology of wheat straw in longitudinal section (**a**–**c**) and cross section (**d**–**f**).

**Figure 3:**
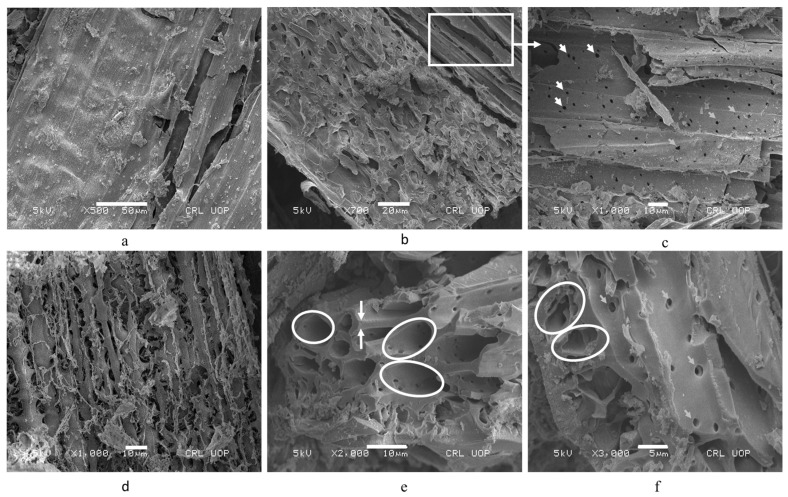
SEM of WSA at varying magnification levels, (**a**–**d**): The fibrous nature of WSA at the macroscopic scale has also been retained at the microscopic level; (**e**,**f**): WSA particles containing micro-perforations, micro-tubules’ of diameter between 0.25 to 2 μm and 2 to 7 μm, respectively.

Microstructural characterization of WSA was performed by conducting scanning electron microscopy (SEM), X-ray diffraction (XRD), and X-ray fluorescence (XRF) to determine micromorphology, crystallographic characteristics, and chemical oxide composition. However, Chapelle’s activity was determined to assess the pozzolanic potential of WSA that might contribute to strength development upon its incorporation in concrete. Wheat straw contains thin and elongated straws that are fibrous at the macroscopic level. Therefore, the micromorphology of both wheat straw and WSA was assessed using SEM using model JSM-5910 JEOL, Japan which are shown in Figure 2 and Figure 3, respectively. Wheat straw contains an external layer (epidermis), the laminated arrangement of layers, and stomata-shaped openings in its longitudinal section (Figure 2a–c). While its cross section contains honeycomb-shaped tubules, microtubules within the aforementioned tubules (Figure 2d,e). The microtubules within the honeycomb tubules in the cross section and (Figure 2d–f) stomata-shaped openings in the longitudinal section, accounted for increased water absorption. Hence, the morphological features account for the low density and high porosity of wheat straws [49].

The fibrous nature of WSA at the macroscopic scale has also been retained at the microscopic level which is evident in Figure 3a–d. WSA particles contain porous and perforated tubules with the size of microperforations and tubules’ diameter ranging from 0.25 to 2 μm and from 2 to 7 μm, respectively (Figure 3e,f). It is believed that the higher water absorption and the low relative density of WSA were attributed to the microperforations and tubules present in ash particles. The SEM micrograph of WSA also shows the lamination of layers having a thickness of approximately 2 µm. Finally, the SEM micrograph of WSA shows flat and elongated particles with a laminated surface while the surface texture appears to be rough and abrasive [30]. Furthermore, published literature [30] suggests that these particles are responsible for the increased water demand of the concrete. It is worth mentioning that the elemental composition and the pozzolanic potential of cement-based composite appear to be influenced by the shape and size of ash particles [50]. Therefore, in future research, it is suggested to investigate the influence of reactivity of cement-based composite upon changing particle size and shapes of WSA.

**Figure 4:**
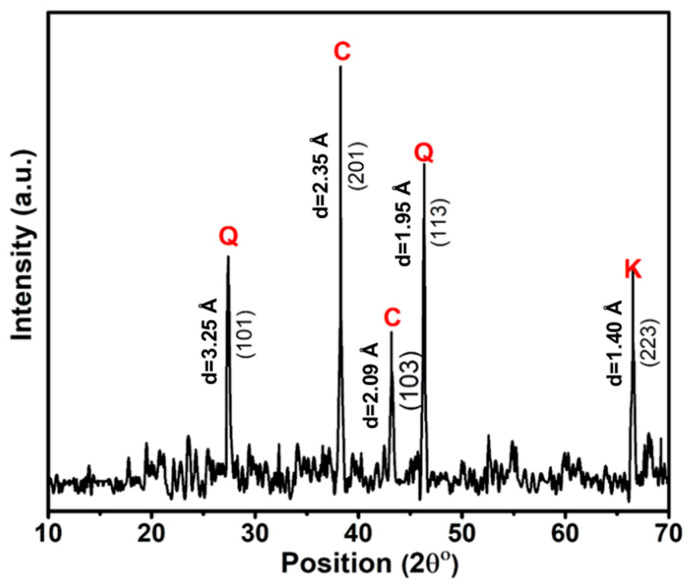
X-ray diffraction pattern of wheat straw ash (Q: Quartz; C: Cristobalite; K: Alumino-Silicate phase).

**Figure 5:**
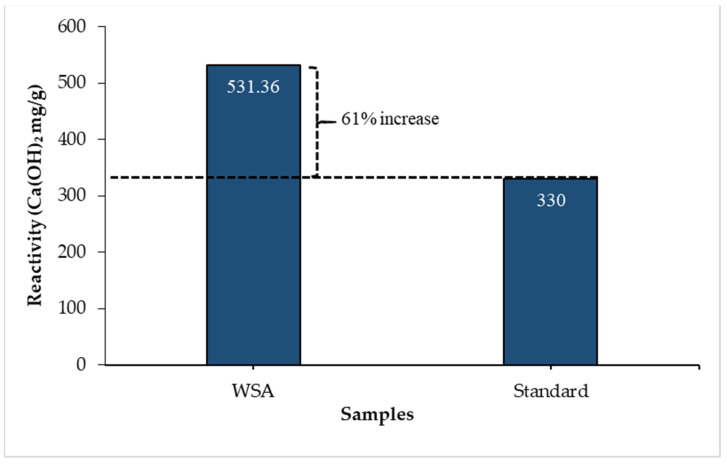
Pozzolanic potential of WSA using Chapelle activity.

The oxide composition of WSA determined by XRF is listed in Table 2. The cumulative concentration of oxides of silica (SiO_2_), alumina (Al_2_O_3_), and iron (Fe_2_O_3_) is 76.22% (>70%), which complies with the chemical requirement of pozzolan as per ASTM C618-15 [51]. The concentration of chemical oxides present in WSA also resembles that cited in the published literature [39,52]. It has been noted that the WSA samples contain a higher proportion of K_2_O, which turns the color of these ashes to dark gray [53]. Crystallographic characteristics were assessed by conducting XRD using diffractometer model JDX-3532 JEOL, Japan, with CuK radiation (1.5418 Å) being operated at 25 mA, 40 kV, and 2θ scan between 05° to 70°. For identifying the crystallographic phases of the WSA specimen, the diffraction patterns were identified using “MATCH Phase Identification v3.1 software” along with the ICDD diffraction pattern. The X-ray diffractometric pattern of wheat straw ash (WSA) is shown in Figure 4. Quartz was identified from sharp diffraction peaks observed at 2θ values of 27.41° (d = 3.25 Å) and 46.50° (d = 1.95 Å). Tetragonal crystals of cristobalite (SiO_2_) were identified at 2θ values of 38.27° (d = 2.35 Å) and 43.19° (d = 2.09 Å). A minor diffraction peak at 66.55° (d = 1.40 Å) was cautiously attributed to alumino-silicate polymorphs. However, qualitative analysis of WSA revealed the amorphous nature of silica. Stutzxnan and Centeno [54] state that the phase diffraction intensities of the peaks are directly proportionate with the concentration of the crystallographic component that produces it, and the difference in the concentration of the components are marked by the difference in intensities of the peaks. The abundance existence of the amorphous silica in WSA can be observed from the diffraction patterns. As the amorphous silica is more desirous than the crystalline silica because of its more reactive nature which helps in accelerating pozzolanic reaction [55]. In this research, the pozzolanic potential of WSA was determined by performing the Chapelle test as per British Standard BS EN 196 [56]. The result of Chapelle activity is shown in Table 3, which displayed the consumption of free lime in terms of milligrams of Ca(OH)_2_ fixed per gram of pozzolan. The pozzolanic activity of WSA found was 497.32 mg/g, i.e., 50.70% greater than that of the standard sample (330 mg/g) which is shown in Figure 5 [57]. The similar extent of the pozzolanic potential of WSA was determined in the literature [39,57]. Therefore, the characterization of WSA revealed that chemical contribution towards strength development might be suspected upon the incorporation of WSA in concrete.

### 2.4. Mix Proportioning and Sample Preparation

The control mix (W0) was formulated using the volumetric method with the design strength of 21 MPa in compression at 28 days. The concrete mixes were designated as W0, W5, W10, W15, and W20 after replacing 0, 5, 10, 15, and 20% of the volume of sand, respectively with WSA. The water absorption of WSA was compensated in mix proportioning to remove the fluctuation of the effective water-cement ratio that might be caused because of WSA’s higher affinity for water. However, water content varies along with the replacement levels but the effective water to cement ratio was affixed 0.5 for all mixes. The mix proportioning of the concrete is shown in Table 4.

### 2.5. Casting and Curing of Specimens

Concrete cube samples (150 × 150 × 150 mm^3^) were cast at the relative humidity and temperature of 90% and 23 ± 4 °C respectively which also comply with the ASTM C 511 [58]. After the molds were removed after 24 ± 1 h, concrete samples were immersed to the specified curing age and the concrete cube samples were tested to determine the various physicomechanical and durability properties up to 90 days of curing age. 

### 2.6. Testing Program

All concrete formulations were assessed for various concrete properties in the fresh and hardened state. Fresh state properties including slump and fresh density were assessed as per ASTM standard C 143 [59] and ASTM standard C138 [60]. The shrinkage response of the concrete is crucial at early stages and influences all other physicomechanical properties, therefore total early shrinkage response was conducted to determine the volumetric stability of the concrete specimens. The shrinkage apparatus (Shwindrine GmBH, Germany), complying with the protocols of linear shrinkage being specified in ASTM C1698 [61], was used to assess the shrinkage response of WSA incorporated mixes. The test samples were wrapped in a polythene sheet to eliminate the loss of water due to evaporation. Relative humidity, temperature, and sensitivity of the mentioned apparatus were kept at 90%, 23 ± 4 °C, and 0.31 µm, respectively. Compressive strength, hardened density, water absorption, and ultrasonic pulse velocity test of concrete specimens were conducted at 7, 28, 56, and 90 days, which comply with BS 1881: Part 116: 1983, ASTM C 642 [62], and ASTM C 597 [63], respectively. The values of the physicomechanical properties represent the average of three tested specimens to reduce the chances of any possible error. For the acid attack test, 150 × 150 × 150 mm^3^ concrete cubes from each mix were cured by water immersion for 28 days, and then oven-dried for 24 h at 105 ± 5 °C, like that reported in the literature [64]. The samples were left to cool until the room temperature has reached after removing them from the oven and the oven-dry weight (W_1_) was determined. Thereafter, the concrete specimens were immersed in a 5% solution of hydrochloric acid and sulfuric acid separately for 28 days. The concrete specimens were oven-dried after removing from the acid solution and their weight was recorded as W_2_. The weight loss of the samples due to the deleterious action of acids was determined using formula given in Equation (1).

(1) $$\mathrm{Weight}\;\mathrm{loss}\;\left(\%\right)=\frac{\left(W_{1}-W_{2}\right)\times 100}{W_{1}}$$

## 3. Discussions of Results

### 3.1. Linear Shrinkage Response

**Figure 6:**
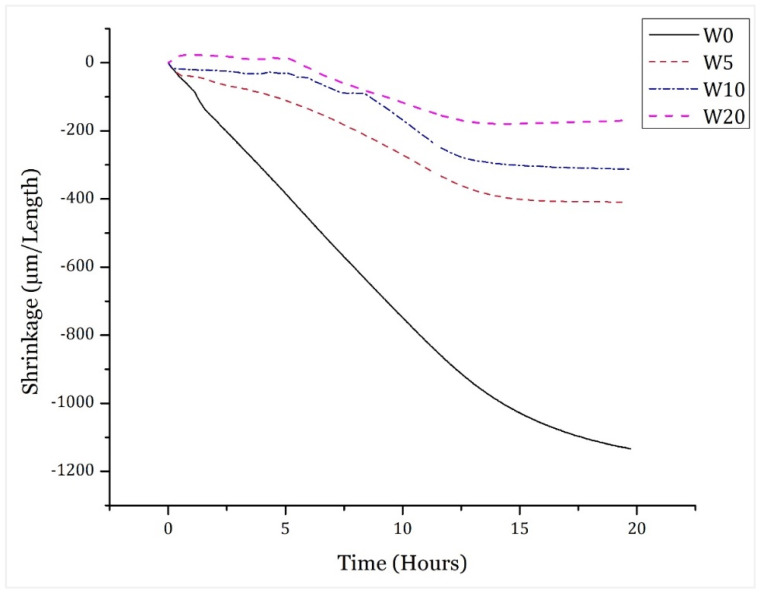
Linear shrinkage response of WSA-incorporated concrete.

The early linear shrinkage response of the cement paste samples containing WSA as partial sand replacement is shown in Figure 6. The decreasing trends of the linear shrinkage of the cement paste were observed upon the incorporation of WSA. In comparison with the control mix (W0), 64.02, 72.39, and 85.36% reduction in shrinkage was observed for the W5, W10, and W20 mixes, respectively. The maximum value of the reduction in shrinkage of cement paste was observed for 20% sand replacement with WSA. All the WSA incorporated mixes have shown a lower shrinkage response comparative to the control mix. Generally, the autogenous shrinkage is caused by the self-desiccation of cement paste matrix upon its hydration, therefore the incorporated WSA act as a water reservoir attributed to its adsorptive nature, which consequently reduces the self-desiccation and the shrinkage [65,66,67]. As the water absorption of WSA was compensated in the mix, therefore the absorbed water within the WSA act as a water reservoir and prevents the cement paste matrix from self-desiccation by fulfilling the demand of water during hydration of cement [65]. Memon et al. [67] utilized CCA as partial sand replacement in concrete and it was observed that the compensated water absorption of CCA reduced the shrinkage response of cement paste due to the adsorptive nature of the incorporated ash. The results of the shrinkage response obtained in this research also comply with that of Bai et al. [68], Ghafoori and Bucholc [69], Singh and Siddique [70], and Kou and Poon [34] in which the shrinkage response of ash incorporated mixes was lower than control concrete. 

### 3.2. Fresh Concrete Properties

**Figure 7:**
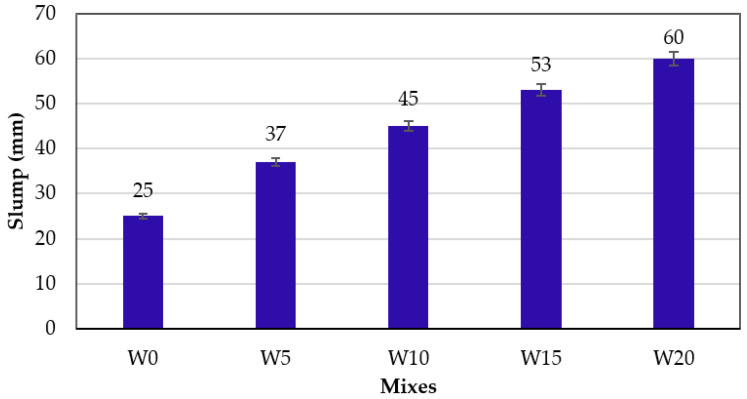
Comparison of the slump of different concrete mixes.

The slump of concrete containing varying WSA content was determined to assess the effect of WSA addition on the workability of concrete. The result of slump values is shown in Figure 7. With the increasing percentage of WSA in concrete, the slump values increased. Slump values of 60 and 25 mm were observed for the W20 and W0 mixes, respectively. The workability of the W20 mix was recorded 2.4 times higher than that of the W0 mix. The higher slump values are due to the lubrication effect caused by the higher amount of water retained in the porous and tubular morphology of WSA as evident in microstructure characterization (Section 2.4). Similar behavior was observed by Kou and Poon [34], in which the complete replacement of recycled aggregate and incremental replacement up to 100% of furnace bottom ash as fine aggregate replacement indicated the rise in slump values as the water required for SSD condition was compensated in the mix. Yung [71] used waste LCD glass as a partial replacement for sand and it was found that the slump values increased from 190 to 210 mm with dilution by 30%. Bai et al. [68] also observed increasing trends of slump values with the increasing percentage of bottom ash. Hence, the concrete slump values increased upon the replacement of WSA due to its higher affinity for water.

**Figure 8:**
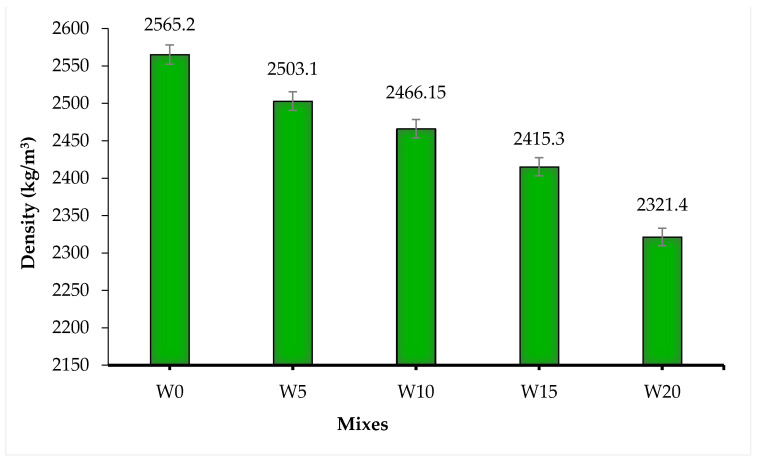
Fresh concrete densities of concrete mix.

Figure 8 shows the results of the fresh concrete density of all mixes. Concrete containing WSA has a lower density than the control mix and a decreasing trend of density was observed with the increasing percentage of WSA. Comparing with the W0 mix, the decrease in the density of fresh concrete for W5, W10, W15, and W20 mixes was found to be 2.47%, 3.86%, 5.84%, and 9.50%, respectively. The porous nature and lower density of WSA, as demonstrated in the previous sections, are responsible for the decrease in the density of WSA incorporated concrete. Aggarwal et al. [72] observed a decrease in the fresh density of concrete containing bottom ash as a fine aggregate replacement, which was due to the lower relative density of bottom ash. Similarly, Yung [71] observed a decrease in the fresh concrete density with increasing waste LCD glass as a fine aggregate replacement at all water-to-binder ratios due to the lower relative density of LCD glass compared to that of sand [71]. Hence, the porous nature and the lower relative density of WSA decreased the fresh concrete density of WSA concrete.

### 3.3. Compressive Strength

**Figure 9:**
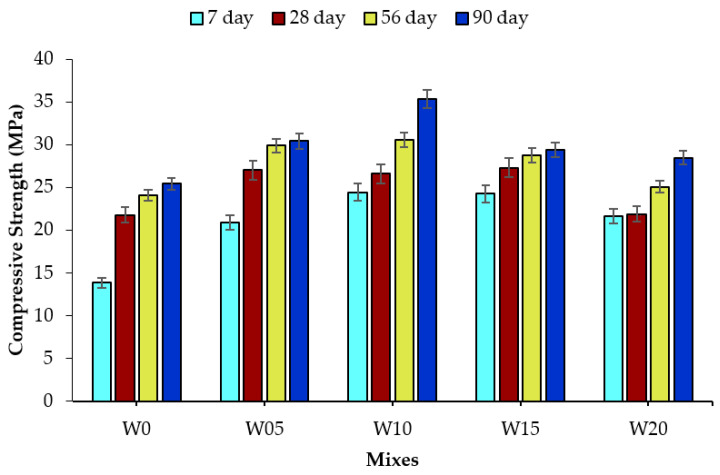
Compressive strength of WSA incorporated concrete mixes.

The compressive strength of concrete containing the specified WSA concentrations was determined to evaluate the effect of WSA addition up to 90 days of curing. The results of compressive strength analysis are shown in Figure 9, which demonstrate that it increased with the age of testing. The compressive strength values of the mixes increased up to 10% incorporation of WSA and then decreased upon further incorporation of WSA but the values remained greater than that of control concrete. In comparison with the control mix, W5, W10, W15, and W20 mixes at 90 days curing showed 19.66, 39.06, 15.69, and 12.07% percent increase in compressive strength, respectively. Among all concrete mixes, the maximum compressive strength of 35.37 MPa was observed for the W10 mix at 90 days. The increased value of compressive strength is believed to be due to the pozzolanic potential demonstrated in the Chapelle test, which caused the densification of the cement paste matrix. The concrete containing 5% WSA (W05 mix) attained maximum compressive strength value of over 27 MPa at 28 days, which exceeds the minimum compressive strength (17 MPa) requirement of structural concrete mentioned in ACI 318-19 [73]. However, the minimum 28 days compressive strength of the W20 mix resulted at 21.87 MPa at 28 days, which can be utilized in foundations and special structural walls made with Grade 60 or 80 reinforcement as per Section 19.2.1.1 of ACI 318-19 [73]. Therefore, all the WSA-incorporated concrete mixes may potentially be used for structural applications as the compressive strength value is greater than 21 MPa at 28 days of curing age. Al-Rawas et al. [74] incorporated incinerated ash as a partial sand replacement in concrete, in which an increase of 28-day compressive strength was observed up to 20% replacement of sand in concrete followed by a decreasing trend upon its further incorporation. Furthermore, published literature [31,75] suggests that the compressive strength of concrete containing waste agro-industrial ashes increased upon their incorporation as a partial fine aggregate replacement. Siddique [29] reported that the compressive strength of concrete increased upon the incorporation of fly ash as a partial fine aggregate replacement, which was attributed to the pozzolanic potential of incorporated ash. Akhras and Alfoul [76] explained the pozzolanic behavior of autoclaved aerated concrete (AAC) containing WSA as partial sand replacement. The ash incorporation activated the pozzolanic reaction by reacting amorphous silica in WSA with calcium hydroxide to produce silicate hydrate gel, which consequently reduced the porosity of concrete by providing nucleation sites for hydration products. Siddique et al. [77] incorporated waste foundry sand as a partial replacement of fine aggregate in concrete and concluded that the mechanical properties of the concrete improved with the increasing curing age [77]. Several other researchers [78] have reported that the incorporation of metallurgical byproducts, foundry sand as a partial replacement of fine aggregate enhances the compressive strength of concrete. In short, the values of the compressive strength of WSA concrete increased up to certain WSA replacement with fine aggregate. Furthermore, the compressive strength increased with the curing age due to the densification of the cement paste matrix as a result of a pozzolanic activity.

### 3.4. Hardened Concrete Density

**Figure 10:**
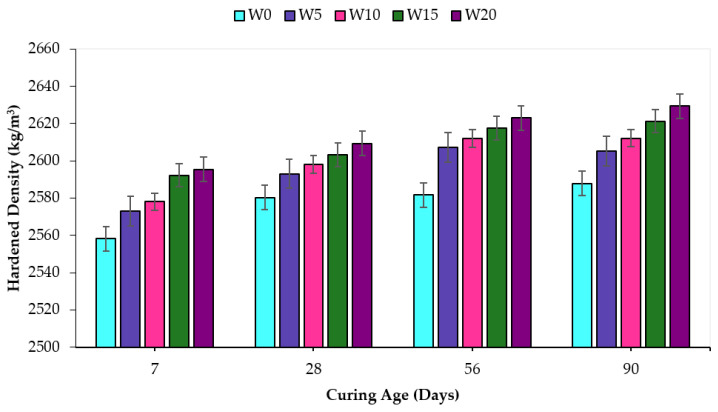
Hardened concrete density containing WSA at varying curing ages.

The hardened density of the concrete formulation with various incorporated percentages of WSA at varying ages of curing is shown in Figure 10. Unlike the fresh concrete density, the hardened density of concrete mix containing WSA increased with all replacement levels and curing ages. The increasing trends of hardened concrete density resemble those of the compressive strength of the W05 and W10 mixes, which are attributed to microstructural densification by the formation of pozzolanic hydrates. However, the hardened density increased for the W15 and W20 mixes with the decreasing values of compressive strength as the densification for the mentioned mixes was believed to occur due to the filler effect. According to published literature [79], the pozzolanic reaction causes the formation of higher-density secondary calcium silicate hydrate (CSH) gel by replacing lower density portlandite formed as a result of hydration. Therefore, the higher values of hardened concrete density have resulted from WSA replacement in W05 and W10 mixes and were due to the pozzolanic activity of WSA. Arif et al. [80] observed similar trends by incorporating the sugarcane bagasse ash as a filler in concrete and observed that the density of the hardened concrete increased at 20% replacement level despite the decrease in compressive strength at the mentioned replacement, thus this increasing density trend was associated with the filler effect of sugarcane bagasse ash at its higher replacement percentage.

**Figure 11:**
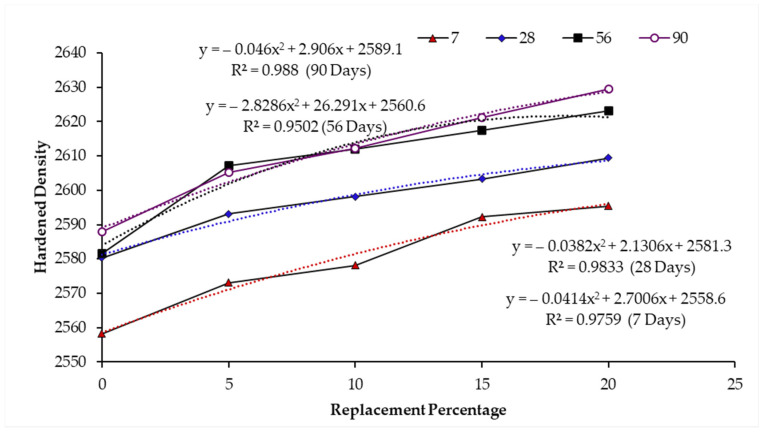
Regression correlation between hardened density and WSA content at various curing ages.

The trend between hardened density and curing age is shown in Figure 11. The regression relation between the increasing density and incorporated WSA content depicts a good correlation between the data points as evident in regression coefficient (R) values of 0.9759, 0.9833, 0.9502, and 0.9880 at 7, 28, 56, and 90 days respectively. Along with the curing ages, the hardened concrete density increased due to pore refinement by clogging microscopic pores attributed to the formation of pozzolanic hydrates [81]. Published literature [29] has witnessed the increased hardened density upon the incorporation of industrial waste ashes as sand replacement in concrete. Siddique [29] revealed that the hardened concrete density and compressive strength increased with the increase in the percentage of fly ash as fine aggregate in the concrete mix. Siddique [77] also noticed the densification of the concrete matrix containing waste foundry sand as a replacement of regular sand due to the formation of pozzolanic hydrates. Therefore, the pozzolanic activity of WSA evident from Chapelle activity seems to impart the densification of the cement paste matrix through the pore refinement process by clogging caused due to pozzolanic hydrates formation [81]. Therefore, the strength development at lower replacement level and higher curing ages was resulted due to pozzolanic potential of WSA, whereas the increase in hardened density for W15 and W20 mixes was attributed to the filler effect. However, it is recommended that scientific investigation should be carried out to further validate this hypothesis.

**Figure 12:**
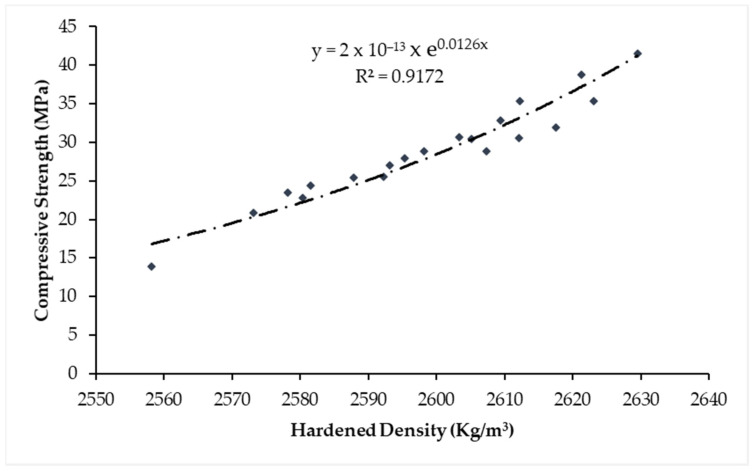
Regression relation between concrete hardened density and compressive strength.

Exponential regression correlation between compressive strength and hardened concrete density is shown in Figure 12. This regression relationship can be used to predict and interpolate the values of compressive strength using hardened density with considerable accuracy as the value of the regression coefficient (R) is 0.91. Therefore, it can be deduced from the performed analysis that hardened density increased with age and upon the replacement of WSA in concrete.

### 3.5. Water Absorption

**Figure 13:**
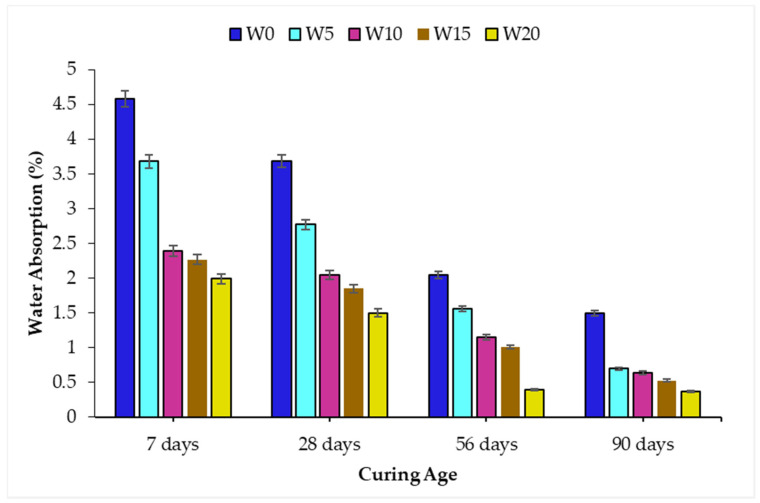
Water absorption of WSA incorporated concrete.

The water absorption of concrete at 7, 28, 56, and 90 days of curing age is shown graphically in Figure 13. The results indicated that upon the incorporation of WSA and along with the testing age, the water absorption of concrete mix formulations decreased. At 7 days, the water absorption of W0 and W20 mixes observed was 4.58% and 1.99% respectively, which was reduced to 1.50% and 0.37% for the mentioned mixes at 90 days. The reduction of water absorption for W0 and W20 mixes between the curing age of 7 and 90 days was recorded at 56.49% and 75.33%, respectively. Minimum water absorption of WSA concrete was recorded at higher percentage replacement. The reduction in water absorption is believed due to the densification of the concrete microstructure of W05 and W10 mixes, which was attributed to the pore refinement by the clogging of microsized capillary voids due to the formation of pozzolanic hydrates [79]. Whereas, the reduction of water absorption for W15 and W20 mixes was observed due to the filler effect contributed by the WSA which is also evident in the published literature [80].

Several published studies [81] have also reported a significant reduction in water absorption at higher curing ages. Arshad et al. [82] assessed the water absorption of self-consolidating concrete containing coal bottom ash as a fine aggregate replacement. The results revealed that the water absorption decreased up to 15% of sand replacement with coal bottom ash. Published literature [83] also reported that the metakaolin and calcined clay incorporation in concrete caused pore refinement of the cement paste matrix which was rapid until the first 14 days. Hence, the incorporation of WSA as partial sand replacement reduced the water absorption with added replacement levels and curing age.

### 3.6. Ultrasonic Pulse Velocity

The concrete quality can be evaluated by determining the velocity of the ultrasonic wave in the cement paste matrix. Densified and enhanced microstructure corresponds to the higher value of UPV because of its lesser transit time. Therefore, the higher the values of UPV, the more durable, compact, impervious and less porous would be the concrete. The ultrasonic pulse velocity values of the WSA concrete formulations are listed in Table 5. The values of UPV increased upon increasing WSA incorporation as fine aggregate replacement in concrete. Despite the lower specific weight of WSA than sand, the values of UPV increased from 5120 m/s to 5750 m/s for the W0 and W20 concrete mixes respectively at 56 days of curing. This might be due to the improvement of concrete microstructure attributed to the reactivity of WSA. Similarly, an increasing trend of UPV with the curing age was observed in the published literature [83]. Sua-iam and Makul [84] suggested that this increase of pulse velocity of self-compacted concrete containing sand replacements with RHA is associated with the degree of densification due to pore refinement and pore reduction in the interfacial transition zone and cement paste matrix. The ultrasonic pulse velocity values obtained from the current study were compared with BIS: 1311-92 [85]. It can be observed that the concrete quality of almost all the mixes can be categorized as excellent.

In literature [83], the researchers correlated UPV values with compressive strength to gauge the compressive strength of concrete. The exponential regression relation between the compressive strength and UPV is stated as follows:

$$\mathrm{Compressive}\;\mathrm{Strength}=A\;\times exp^{\left(B\left(\mathrm{UPV}\right)\right)}$$

**Figure 14:**
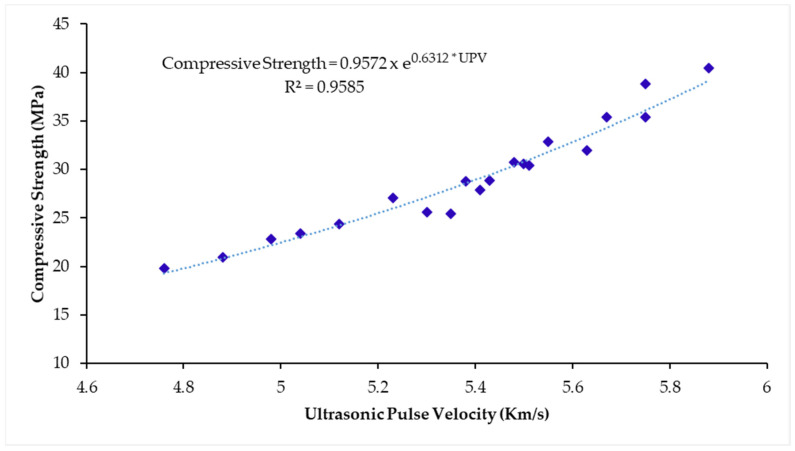
Regression relation between compressive strength and UPV of WSA incorporated mixes.

While A and B are empirical constants. The regression relation between the mentioned properties was determined which is shown in Figure 14. The regression coefficient (R) was 0.9783, 0.9906, 0.9905, and 0.9865 at 7, 28, 56, and 90 days of curing age respectively, depicting a good connection between regression curve and data points.

**Figure 15:**
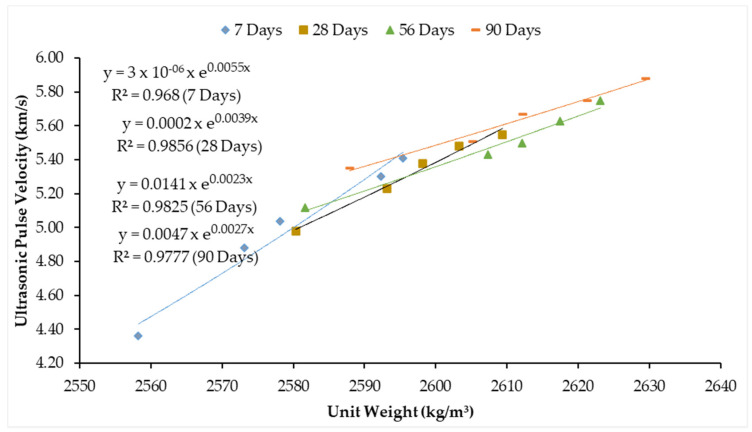
Relation between hardened density and UPV of concrete.

It is well-known that the travel time or velocity of an ultrasonic wave is affected by the nature of the propagating medium. Therefore, the density of the medium is of prime importance. Hence, the relation between unit weight and UPV is explicitly shown in Figure 15. The correlation coefficients for unit weight and ultrasonic pulse velocity are 0.968, 0.9856, 0.9825, and 0.9777 at 7, 28, 56, and 90 days respectively. Therefore, good relation is exhibited between the regression curve and data points.

### 3.7. Response in Acidic Media

**Figure 16:**
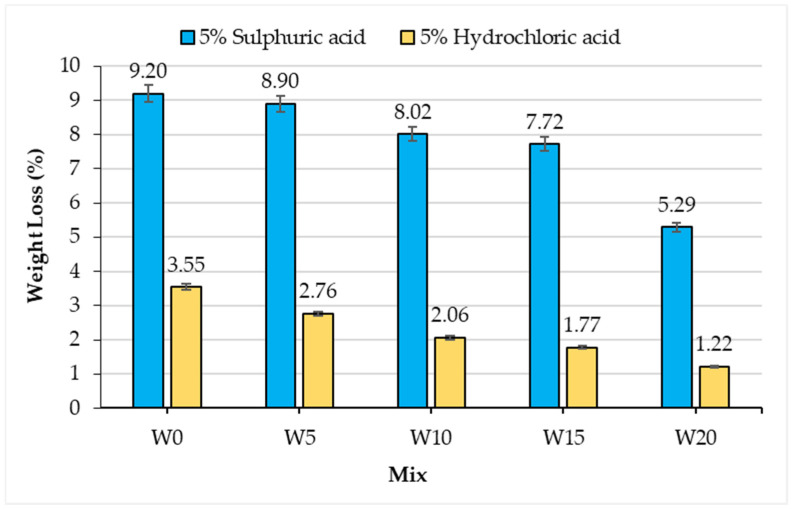
Deterioration of concrete mixes in terms of weight loss after exposure to the acid solution.

Concrete has an alkaline nature which is vulnerable to acid attack. The binding phase (silicate phases) gets disintegrated while in contact with strong acids. Therefore, the performance of concrete formulations in a severe acidic environment was evaluated by keeping the concrete samples in hydrochloric acid and sulfuric acid for 28 days. After acid exposure, the weight loss results of control and WSA replaced concrete was determined and the results are shown in Figure 16. The weight loss decreases with the increase of replacement percentages for both sulfuric and hydrochloric acids. As discussed earlier, the concrete water absorption at any specific age reduced with the increase in the replacement percentage of fine aggregate with WSA. The same analogy applies for acid medium: with the increase in WSA percentage in the concrete mix, the less acid will be absorbed. Hence, the lesser deterioration resulted in a minimal weight loss. For sulfuric acid, minimum and maximum weight loss of 5.29% and 9.20% were observed for W20 and W0 concrete mixes, respectively. Similarly, for exposure to hydrochloric acid, minimum and maximum weight loss of 1.22% and 3.55% were observed for W20 and W0 concrete mix, respectively. These trends revealed that the deterioration caused by sulfuric acid is much more adverse than that of hydrochloric acid. Similarly, published literature [75] also suggests the higher value of weight loss due to sulphuric acid is because of delayed ettringite formation (DEF) which causes excessive expansion and disruption of the hardened cement paste. In short, WSA incorporated concrete offered better resistance toward acidic environments.

### 3.8. Thermogravimetric Analysis (TGA)

**Figure 17:**
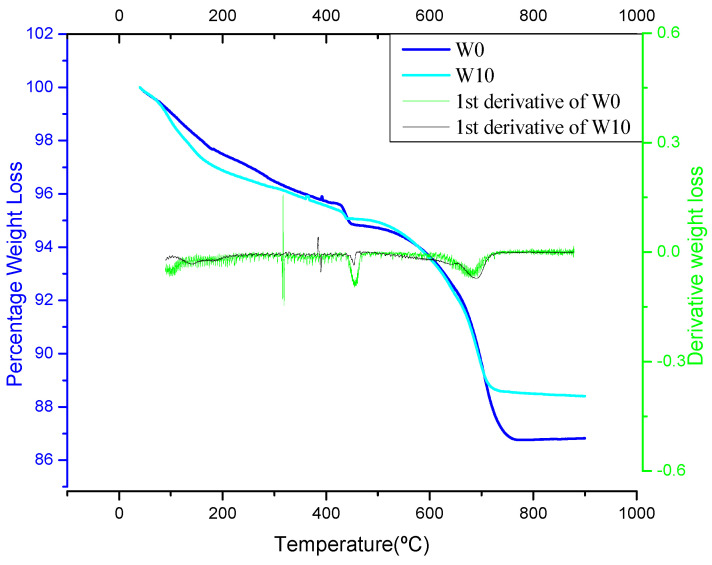
Thermogravimetric analysis and derivative weight loss curves of W0 and W10 mixes.

The pozzolanic nature of WSA in concrete mixes was determined by the thermogravimetric analysis. For the ascribed reason, the TGA of the control mix (W0) and the mix having maximum compressive strength (W10) was determined at the curing age of 90 days. The weight loss and TGA curve for the W0 and W10 mixes are shown in Table 6 and Figure 17, respectively. Three weight-loss regions were identified. The first weight loss observed in the temperature region between 110 to 300 °C which is mainly associated with dehydration of C-S-H. The second weight loss occurred in 450–550 °C due to the dehydroxylation of portlandite while the third weight loss region within the temperature range of 750–900 °C is related to the decarbonization of calcium carbonate [86,87]. Referring to the first region, the WSA concrete mix (W10) showed a higher weight loss (18.64%) than the control mix (W0) 18.19%. Therefore, a higher extent of hydration occurred than in control concrete. In the second region, the weight loss for the concrete containing WSA as a fine aggregate replacement (W10) experienced 1.88% weight loss, while concrete experienced 3.54% weight loss. Lower weight loss in this region shows that Ca(OH)_2_ was consumed by the WSA, confirming the pozzolanic activity. Similar trends of lower weight loss in the second region were observed in the published literature [87,88], which depicted the extent of higher pozzolanic activity with a lower concentration of portlandite. The third weight loss region is associated with the decarbonization of cement paste, which can be used as an indicator of hydration degree at the initial stage (24 h) [86]. The lower weight loss value of the W0 (0.11%) mix than W10 (0.16%) in the decarbonization region is indicative of a higher early hydration rate of control concrete in the form of CSH gel and portlandite production. Hence, TGA results validate the pozzolanic potential of WSA as fine aggregate-based material. It can therefore be deduced that, due to the pozzolanic potential of WSA, concrete resulted in higher compressive strength and hardened density, whereas the lower water absorption values comparative to control concrete.

## 4. Conclusions

Environmentally friendly disposal of WSA by incorporating it as partial fine aggregate replacement in concrete has been proposed in this research. A few of the prime findings of this research are:

(a) Macro and microstructural characterization results of WSA revealed that it is pozzolanic in nature, well-graded with a fineness modulus of 2.76, free from organic impurities, and compliant with the ASTM C33 gradation requirement along with porous morphology due to the presence of tubules and microperforations.
(b) Increasing slump values were observed with increased WSA replacement levels. This trend was predictable because of the lubrication effect of excessive availability of water absorbed by WSA, hence increased the fluidity of WSA concrete mixes. A maximum value of 85.36% reduction in total shrinkage response at 20% incorporation of WSA might be due to the readily available absorbed water that minimizes the self-desiccation of the cement paste matrix. The maximum decrease in fresh density was 9.5% compared to the control mix.
(c) Increasing compressive strength values were attained with both increases in the age of testing and the WSA replacement percentage in concrete mixes. This increase was attributed due to the pozzolanic potential. At 28 days curing age, the compressive strength of all concrete formulations comply with the requirement of structural concrete as the minimal compressive strength of the mixes was higher than 17 MPa being specified in ACI 318, therefore it can potentially be used in various structural configurations.
(d) The hardened concrete density of WSA incorporated concrete mixes increased along with the replacement percentages as a result of a pozzolanic reaction and filler effect. The water absorption of the concrete decreased upon the replacement of WSA due to suspected pore refinement by clogging of capillary voids which occurred due to synergic action of pozzolanic reaction and filler effect caused by WSA.
(e) Like trends of hardened density, the pulse velocity values increased at all testing ages and replacement percentages. Based on the values of UPV, all WSA incorporated mixes fall in the category of excellent quality concrete. Therefore, higher UPV values show that the quality of the cement paste matrix has been enhanced upon the incorporation of WSA in concrete.
(f) Weight loss of concrete containing WSA decreased with the increase in replacement percentage under the action of sulfuric acid and hydrochloric acid. The adverse effects of acids on concrete reduced with the addition of WSA due to densification in the microstructure. Therefore, the resistance toward deleterious agents increased with the increase of WSA incorporation in concrete.
(g) The incorporation of WSA in the W10 mix has shown the lower value of weight loss in the second region which corresponds to the lower percentage of portlandite than that in control concrete. Thus, the WSA also shows pozzolanic activity when used as fine aggregate replacement in concrete.

Conclusively, environmentally friendly incorporation of WSA provides a solution for ash disposal and subdues ecological depletion, especially air pollution, which poses a serious hazard to human health. For the construction industry, it would help in the conservation of natural aggregate resources by providing a feasible source of raw materials.

## Figures and Tables

**Table 1:** Relative density, water absorption of sand, WSA, and coarse aggregate.

	Sand	Wheat Straw Ash	Coarse Aggregate
Water absorption (%)	2.40	13.61	0.80
Relative density (Ratio)	2.62	1.89	2.63

**Table 2:** Chemical composition of WSA.

Oxide Composition	Percentage
SiO_2_	71.98
Fe_2_O_3_	1.94
Al_2_O_3_	2.30
CaO	3.05
MgO	2.58
P_2_O_5_	1.27
K_2_O	9.12
Na_2_O	2.7
TiO_2_	0.04
MnO	0.03
LOI	4.99
(SiO_2_+Al_2_O_3_+ Fe_2_O_3_)	76.22
Ash content	2.3

**Table 3:** Chapelle activity in WSA and cement.

Filtrate (mL)	Titration HCL Volume for Cement (V_1_)	Titration HCL Volume for WSA (V_2_)	Chappelle Activity (Ca(OH)₂ mg/g) ((V_1_ – V_2_)/V_1_) * 2642.86
25	18.6	15.10	497.32

**Table 4:** Mix proportioning of WSA incorporated concrete.

Mix Design	Cement (kg/m^3^)	Water to Cement Ratio	Sand (kg/m^3^)	Wheat Straw Ash (kg/m^3^)	Coarse Aggregate (kg/m^3^)	Water (kg/m^3^)
W0(CM)	426	0.50	911.22	0	1146.14	212.29
W5	426	0.50	865.66	32.87	1146.14	216.76
W10	426	0.50	820.00	65.73	1146.14	225.71
W15	426	0.50	774.54	98.60	1146.14	239.13
W20	426	0.50	728.98	131.47	1146.14	257.02

**Table 5:** UPV of the WSA concrete and the concrete quality grading specified in BIS.

Mix	Ultrasonic Pulse Velocity (m/s)				Concrete Quality Grading as per BIS 13311-92-Part-I	
	7 d	28 d	56 d	90 d	Pulse Velocity(m/s)	Concrete quality grading
W0	4360	4980	5120	5350	Above 4500	Excellent
W5	4880	5230	5430	5510	3500–4500	Good
W10	5040	5380	5500	5670	3000–3500	Medium
W15	5300	5480	5630	5750	Less than 3000	Doubtful
W20	5410	5550	5750	5880		

**Table 6:** Weight loss values of W0 and W10 mortar at 90 days [86].

Concrete Specimens	Weight Loss (%)			Weight Loss with Respect to Total Weight Loss (%)		
	Stage 1	Stage 2	Stage 3	Stage 1	Stage 2	Stage 3
W0	2.22	0.47	0.11	18.19	3.54	0.83
W10	2.40	0.23	0.16	18.64	1.88	1.34

Note: Stage 1: dehydration occurred due water loss from C–S–H; Stage 2: Dehydroxylation of portlandite; Stage 3: Decarbonation of calcium carbonate.

## Data Availability

The data used to support the findings of this study are included within the article.
