# Correction: Memon et al. Incorporation of Wheat Straw Ash as Partial Sand Replacement for Production of Eco-Friendly Concrete. *Materials* 2021, *14*, 2078

**DOI:** 10.3390/ma19132759

**Published:** 2026-06-29

**Authors:** Shazim Ali Memon, Usman Javed, Muhammad Haris, Rao Arsalan Khushnood, Jong Kim

**Affiliations:** 1 Department of Civil and Environmental Engineering, School of Engineering and Digital Sciences, Nazarbayev University, Astana 010000, Republic of Kazakhstan; jong.kim@nu.edu.kz; 2 School of Civil and Mechanical Engineering, Curtin University, Perth, WA 6102, Australia; usman.javed1@postgrad.curtin.edu.au; 3 Department of Civil Engineering, COMSATS Institute of Information and Technology, Abbottabad 22060, Pakistan; mharis8408@gmail.com; 4 NUST Institute of Civil Engineering, National University of Sciences and Technology, Islamabad 44000, Pakistan; arsalan.khushnood@nice.nust.edu.pk

In the original publication [1], there was a mistake in Figure 4. The corrected Figure 4 and its caption appears below:

Correspondingly, the citation of Figure 4 in Section 2.3. Microstructural Characterization, paragraph 3 has been corrected. The corrected paragraph reads as follows:

The oxide composition of WSA determined by XRF is listed in Table 2. The cumulative concentration of oxides of silica (SiO_2_), alumina (Al_2_O_3_), and iron (Fe_2_O_3_) is 76.22% (>70%), which complies with the chemical requirement of pozzolan as per ASTM C618-15 [51]. The concentration of chemical oxides present in WSA also resembles that cited in the published literature [39,52]. It has been noted that the WSA samples contain a higher proportion of K_2_O, which turns the color of these ashes to dark gray [53]. Crystallographic characteristics were assessed by conducting XRD using diffractometer model JDX-3532 JEOL, Japan, with CuK radiation (1.5418 Å) being operated at 25 mA, 40 kV, and 2θ scan between 05° to 70°. For identifying the crystallographic phases of the WSA specimen, the diffraction patterns were identified using “MATCH Phase Identification v3.1 software” along with the ICDD diffraction pattern. The X-ray diffractometric pattern of wheat straw ash (WSA) is shown in Figure 4. Quartz was identified from sharp diffraction peaks observed at 2θ values of 27.41° (d = 3.25 Å) and 46.50° (d = 1.95 Å). Tetragonal crystals of cristobalite (SiO_2_) were identified at 2θ values of 38.27° (d = 2.35 Å) and 43.19° (d = 2.09 Å). A minor diffraction peak at 66.55° (d = 1.40 Å) was cautiously attributed to alumino-silicate polymorphs. However, qualitative analysis of WSA revealed the amorphous nature of silica. Stutzxnan and Centeno [54] state that the phase diffraction intensities of the peaks are directly proportionate with the concentration of the crystallographic component that produces it, and the difference in the concentration of the components are marked by the difference in intensities of the peaks. The abundance existence of the amorphous silica in WSA can be observed from the diffraction patterns. As the amorphous silica is more desirous than the crystalline silica because of its more reactive nature which helps in accelerating pozzolanic reaction [55]. In this research, the pozzolanic potential of WSA was determined by performing the Chapelle test as per British Standard BS EN 196 [56]. The result of Chapelle activity is shown in Table 3, which displayed the consumption of free lime in terms of milligrams of Ca(OH)_2_ fixed per gram of pozzolan. The pozzolanic activity of WSA found was 497.32 mg/g, i.e., 50.70% greater than that of the standard sample (330 mg/g) which is shown in Figure 5 [57]. The similar extent of the pozzolanic potential of WSA was determined in the literature [39,57]. Therefore, the characterization of WSA revealed that chemical contribution towards strength development might be suspected upon the incorporation of WSA in concrete.

The authors state that the scientific conclusions are unaffected. This correction was approved by the Academic Editor. The original publication has also been updated.

## Figures and Tables

**Figure 4:**
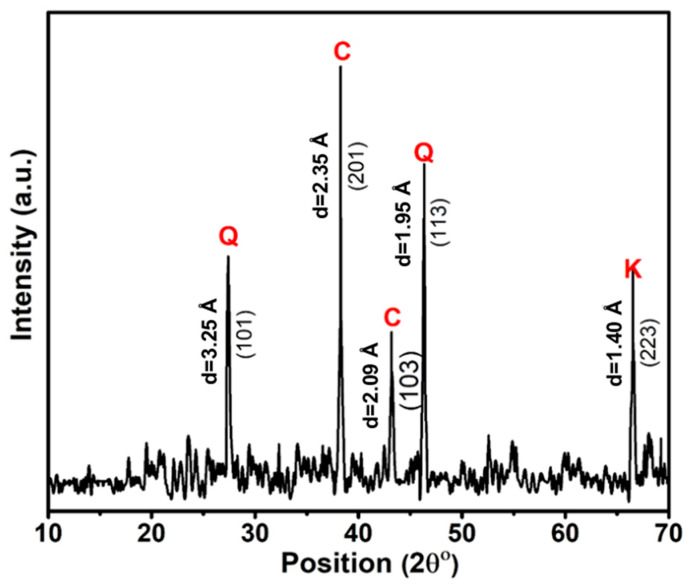
X-ray diffraction pattern of wheat straw ash (Q: Quartz; C: Cristobalite; K: Alumino-Silicate phase).
